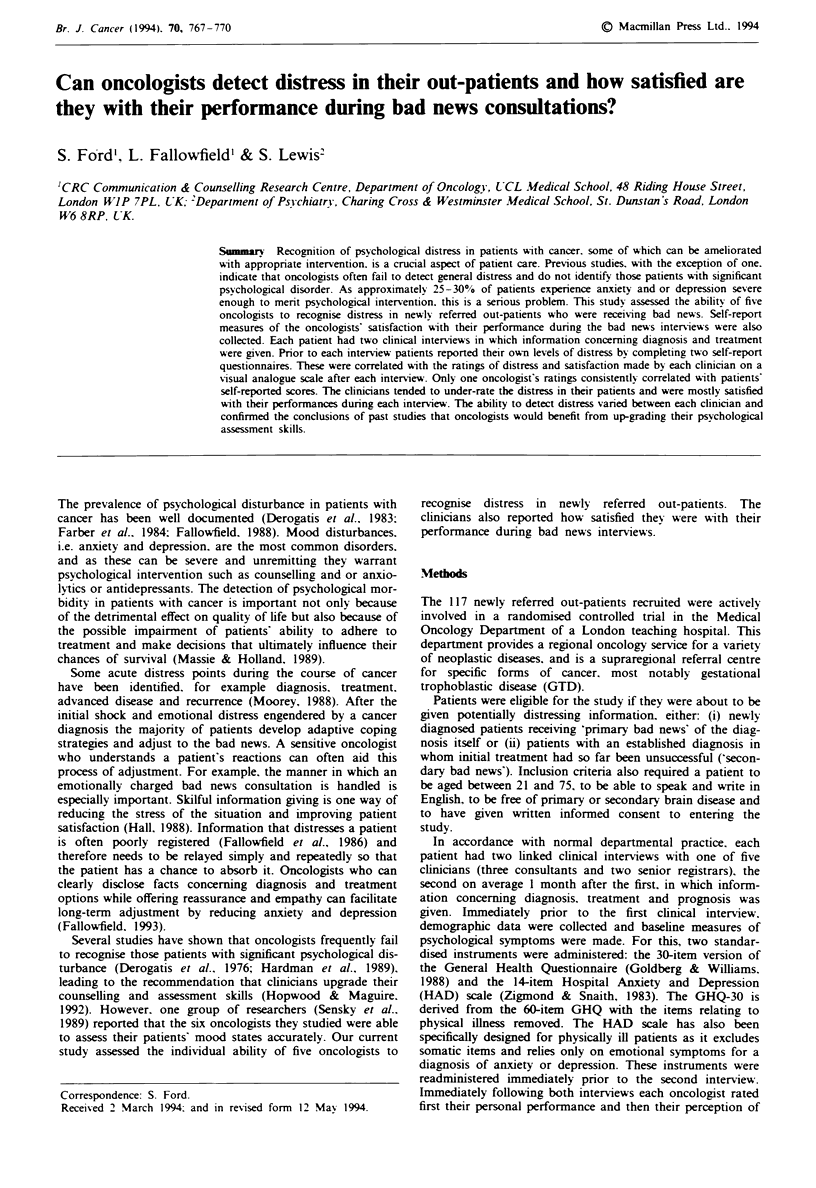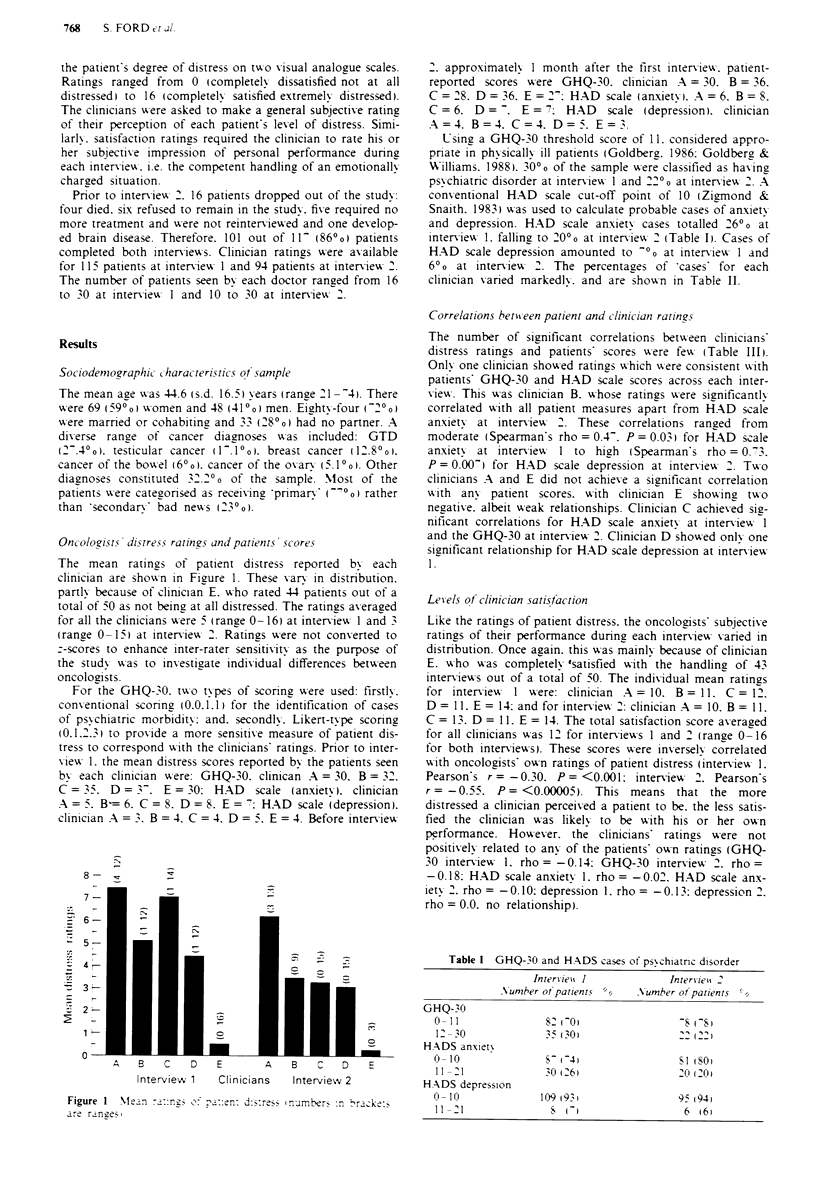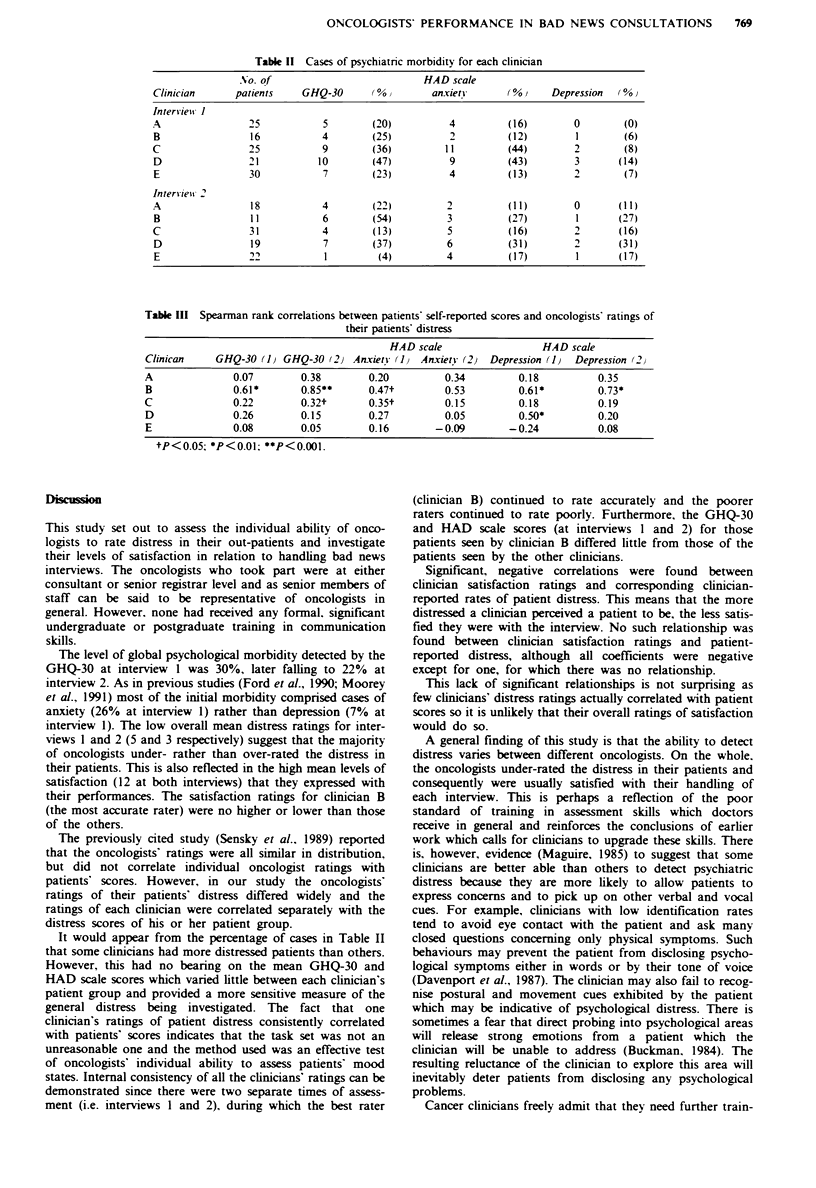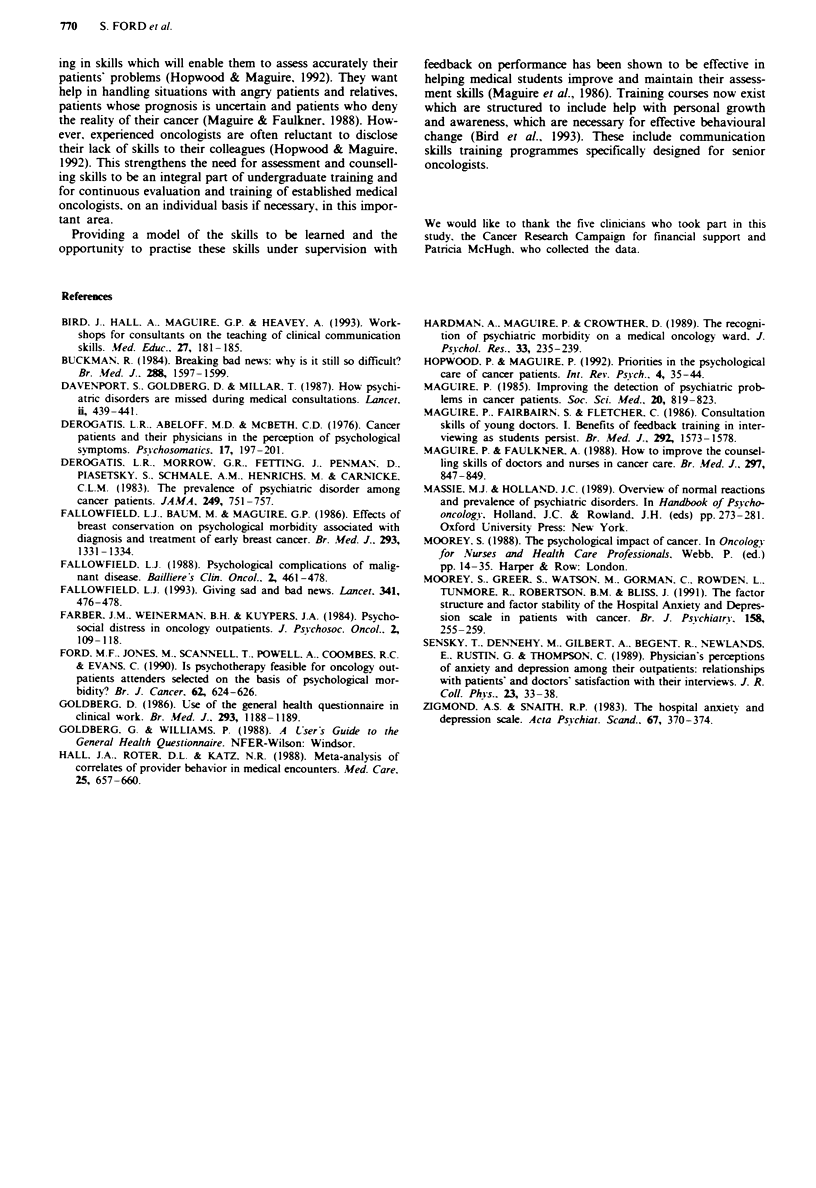# Can oncologists detect distress in their out-patients and how satisfied are they with their performance during bad news consultations?

**DOI:** 10.1038/bjc.1994.393

**Published:** 1994-10

**Authors:** S. Ford, L. Fallowfield, S. Lewis

**Affiliations:** Department of Oncology, UCL Medical School, London, UK.

## Abstract

Recognition of psychological distress in patients with cancer, some of which can be ameliorated with appropriate intervention, is a crucial aspect of patient care. Previous studies, with the exception of one, indicate that oncologists often fail to detect general distress and do not identify those patients with significant psychological disorder. As approximately 25-30% of patients experience anxiety and/or depression severe enough to merit psychological intervention, this is a serious problem. This study assessed the ability of five oncologists to recognise distress in newly referred out-patients who were receiving bad news. Self-report measures of the oncologists' satisfaction with their performance during the bad news interviews were also collected. Each patient had two clinical interviews in which information concerning diagnosis and treatment were given. Prior to each interview patients reported their own levels of distress by completing two self-report questionnaires. These were correlated with the ratings of distress and satisfaction made by each clinician on a visual analogue scale after each interview. Only one oncologist's ratings consistently correlated with patients' self-reported scores. The clinicians tended to under-rate the distress in their patients and were mostly satisfied with their performances during each interview. The ability to detect distress varied between each clinician and confirmed the conclusions of past studies that oncologists would benefit from up-grading their psychological assessment skills.


Br. J. Cancer (1994), 70, 767-770 © Macmillan Press Ltd., 1994

### Can oncologists detect distress in their out-patients and how satisfied are they with their performance during bad news consultations?

S. Ford[1], L. Fallowfield[1] & S. Lewis[2]

[1]CRC Communication & Counselling Research Centre, Department of Oncology, UCL Medical School, 48 Riding House Street, London W1P 7PL, UK; [2]Department of Psychiatry, Charing Cross & Westminster Medical School, St. Dunstan's Road, London W6 8RP, UK.

**Summary** Recognition of psychological distress in patients with cancer, some of which can be ameliorated with appropriate intervention, is a crucial aspect of patient care. Previous studies, with the exception of one, indicate that oncologists often fail to detect general distress and do not identify those patients with significant psychological disorder. As approximately 25-30% of patients experience anxiety and or depression severe enough to merit psychological intervention, this is a serious problem. This study assessed the ability of five oncologists to recognise distress in newly referred out-patients who were receiving bad news. Self-report measures of the oncologists' satisfaction with their performance during the bad news interviews were also collected. Each patient had two clinical interviews in which information concerning diagnosis and treatment were given. Prior to each interview patients reported their own levels of distress by completing two self-report questionnaires. These were correlated with the ratings of distress and satisfaction made by each clinician on a visual analogue scale after each interview. Only one oncologist's ratings consistently correlated with patients' self-reported scores. The clinicians tended to under-rate the distress in their patients and were mostly satisfied with their performances during each interview. The ability to detect distress varied between each clinician and confirmed the conclusions of past studies that oncologists would benefit from up-grading their psychological assessment skills.

The prevalence of psychological disturbance in patients with cancer has been well documented (Derogatis *et al.*, 1983; Farber *et al.*, 1984; Fallowfield, 1988). Mood disturbances, i.e. anxiety and depression, are the most common disorders, and as these can be severe and unremitting they warrant psychological intervention such as counselling and or anxiolytics or antidepressants. The detection of psychological morbidity in patients with cancer is important not only because of the detrimental effect on quality of life but also because of the possible impairment of patients' ability to adhere to treatment and make decisions that ultimately influence their chances of survival (Massie & Holland, 1989).

Some acute distress points during the course of cancer have been identified, for example diagnosis, treatment, advanced disease and recurrence (Moorey, 1988). After the initial shock and emotional distress engendered by a cancer diagnosis the majority of patients develop adaptive coping strategies and adjust to the bad news. A sensitive oncologist who understands a patient's reactions can often aid this process of adjustment. For example, the manner in which an emotionally charged bad news consultation is handled is especially important. Skilful information giving is one way of reducing the stress of the situation and improving patient satisfaction (Hall, 1988). Information that distresses a patient is often poorly registered (Fallowfield *et al.*, 1986) and therefore needs to be relayed simply and repeatedly so that the patient has a chance to absorb it. Oncologists who can clearly disclose facts concerning diagnosis and treatment options while offering reassurance and empathy can facilitate long-term adjustment by reducing anxiety and depression (Fallowfield, 1993).

Several studies have shown that oncologists frequently fail to recognise those patients with significant psychological disturbance (Derogatis *et al.*, 1976; Hardman *et al.*, 1989), leading to the recommendation that clinicians upgrade their counselling and assessment skills (Hopwood & Maguire, 1992). However, one group of researchers (Sensky *et al.*, 1989) reported that the six oncologists they studied were able to assess their patients' mood states accurately. Our current study assessed the individual ability of five oncologists to recognise distress in newly referred out-patients. The clinicians also reported how satisfied they were with their performance during bad news interviews.

Correspondence: S. Ford.
Received 2 March 1994; and in revised form 12 May 1994.

### Methods

The 117 newly referred out-patients recruited were actively involved in a randomised controlled trial in the Medical Oncology Department of a London teaching hospital. This department provides a regional oncology service for a variety of neoplastic diseases, and is a supraregional referral centre for specific forms of cancer, most notably gestational trophoblastic disease (GTD).

Patients were eligible for the study if they were about to be given potentially distressing information, either: (i) newly diagnosed patients receiving 'primary bad news' of the diagnosis itself or (ii) patients with an established diagnosis in whom initial treatment had so far been unsuccessful ('secondary bad news'). Inclusion criteria also required a patient to be aged between 21 and 75, to be able to speak and write in English, to be free of primary or secondary brain disease and to have given written informed consent to entering the study.

In accordance with normal departmental practice, each patient had two linked clinical interviews with one of five clinicians (three consultants and two senior registrars), the second on average 1 month after the first, in which information concerning diagnosis, treatment and prognosis was given. Immediately prior to the first clinical interview, demographic data were collected and baseline measures of psychological symptoms were made. For this, two standardised instruments were administered: the 30-item version of the General Health Questionnaire (Goldberg & Williams, 1988) and the 14-item Hospital Anxiety and Depression (HAD) scale (Zigmond & Snaith, 1983). The GHQ-30 is derived from the 60-item GHQ with the items relating to physical illness removed. The HAD scale has also been specifically designed for physically ill patients as it excludes somatic items and relies only on emotional symptoms for a diagnosis of anxiety or depression. These instruments were readministered immediately prior to the second interview. Immediately following both interviews each oncologist rated first their personal performance and then their perception of the patient's degree of distress on two visual analogue scales. Ratings ranged from 0 (completely dissatisfied not at all distressed) to 16 (completely satisfied extremely distressed). The clinicians were asked to make a general subjective rating of their perception of each patient's level of distress. Similarly, satisfaction ratings required the clinician to rate his or her subjective impression of personal performance during each interview, i.e. the competent handling of an emotionally charged situation.

Prior to interview 2, 16 patients dropped out of the study: four died, six refused to remain in the study, five required no more treatment and were not reinterviewed and one developed brain disease. Therefore, 101 out of 117 (86%) patients completed both interviews. Clinician ratings were available for 115 patients at interview 1 and 94 patients at interview 2. The number of patients seen by each doctor ranged from 16 to 30 at interview 1 and 10 to 30 at interview 2.

### Results

*Sociodemographic characteristics of sample*

The mean age was 44.6 (s.d. 16.5) years (range 21–74). There were 69 (59%) women and 48 (41%) men. Eighty-four (72%) were married or cohabiting and 33 (28%) had no partner. A diverse range of cancer diagnoses was included: GTD (27.4%), testicular cancer (17.1%), breast cancer (12.8%), cancer of the bowel (6%), cancer of the ovary (5.1%). Other diagnoses constituted 32.2% of the sample. Most of the patients were categorised as receiving 'primary' (77%) rather than 'secondary' bad news (23%).

*Oncologists' distress ratings and patients' scores*

The mean ratings of patient distress reported by each clinician are shown in Figure 1. These vary in distribution, partly because of clinician E, who rated 44 patients out of a total of 50 as not being at all distressed. The ratings averaged for all the clinicians were 5 (range 0–16) at interview 1 and 3 (range 0–15) at interview 2. Ratings were not converted to z-scores to enhance inter-rater sensitivity as the purpose of the study was to investigate individual differences between oncologists.

For the GHQ-30, two types of scoring were used: firstly, conventional scoring (0,0,1,1) for the identification of cases of psychiatric morbidity; and, secondly, Likert-type scoring (0,1,2,3) to provide a more sensitive measure of patient distress to correspond with the clinicians' ratings. Prior to interview 1, the mean distress scores reported by the patients seen by each clinician were: GHQ-30, clinican A = 30, B = 32, C = 35, D = 37, E = 30; HAD scale (anxiety), clinician A = 5, B = 6, C = 8, D = 8, E = 7; HAD scale (depression), clinician A = 3, B = 4, C = 4, D = 5, E = 4. Before interview 2, approximately 1 month after the first interview, patient-reported scores were GHQ-30, clinician A = 30, B = 36, C = 28, D = 36, E = 27; HAD scale (anxiety), A = 6, B = 8, C = 6, D = 7, E = 7; HAD scale (depression), clinician A = 4, B = 4, C = 4, D = 5, E = 3.

**Figure 1** Mean ratings of patient distress (numbers in brackets are ranges).

Using a GHQ-30 threshold score of 11, considered appropriate in physically ill patients (Goldberg, 1986; Goldberg & Williams, 1988), 30% of the sample were classified as having psychiatric disorder at interview 1 and 22% at interview 2. A conventional HAD scale cut-off point of 10 (Zigmond & Snaith, 1983) was used to calculate probable cases of anxiety and depression. HAD scale anxiety cases totalled 26% at interview 1, falling to 20% at interview 2 (Table I). Cases of HAD scale depression amounted to 7% at interview 1 and 6% at interview 2. The percentages of 'cases' for each clinician varied markedly, and are shown in Table II.

*Correlations between patient and clinician ratings*

The number of significant correlations between clinicians' distress ratings and patients' scores were few (Table III). Only one clinician showed ratings which were consistent with patients' GHQ-30 and HAD scale scores across each interview. This was clinician B, whose ratings were significantly correlated with all patient measures apart from HAD scale anxiety at interview 2. These correlations ranged from moderate (Spearman's rho = 0.47, $P = 0.03$) for HAD scale anxiety at interview 1 to high (Spearman's rho = 0.73, $P = 0.007$) for HAD scale depression at interview 2. Two clinicians A and E did not achieve a significant correlation with any patient scores, with clinician E showing two negative, albeit weak relationships. Clinician C achieved significant correlations for HAD scale anxiety at interview 1 and the GHQ-30 at interview 2. Clinician D showed only one significant relationship for HAD scale depression at interview 1.

*Levels of clinician satisfaction*

Like the ratings of patient distress, the oncologists' subjective ratings of their performance during each interview varied in distribution. Once again, this was mainly because of clinician E, who was completely satisfied with the handling of 43 interviews out of a total of 50. The individual mean ratings for interview 1 were: clinician A = 10, B = 11, C = 12, D = 11, E = 14; and for interview 2: clinician A = 10, B = 11, C = 13, D = 11, E = 14. The total satisfaction score averaged for all clinicians was 12 for interviews 1 and 2 (range 0–16 for both interviews). These scores were inversely correlated with oncologists' own ratings of patient distress (interview 1, Pearson's $r = -0.30$, $P = <0.001$; interview 2, Pearson's $r = -0.55$, $P = <0.00005$). This means that the more distressed a clinician perceived a patient to be, the less satisfied the clinician was likely to be with his or her own performance. However, the clinicians' ratings were not positively related to any of the patients' own ratings (GHQ-30 interview 1, rho = −0.14; GHQ-30 interview 2, rho = −0.18; HAD scale anxiety 1, rho = −0.02, HAD scale anxiety 2, rho = −0.10; depression 1, rho = −0.13; depression 2, rho = 0.0, no relationship).

**Table I** GHQ-30 and HADS cases of psychiatric disorder

| | *Interview 1* Number of patients % | *Interview 2* Number of patients % |
|---|---|---|
| GHQ-30 | | |
| 0–11 | 82 (70) | 78 (78) |
| 12–30 | 35 (30) | 22 (22) |
| HADS anxiety | | |
| 0–10 | 87 (74) | 81 (80) |
| 11–21 | 30 (26) | 20 (20) |
| HADS depression | | |
| 0–10 | 109 (93) | 95 (94) |
| 11–21 | 8 (7) | 6 (6) |

**Table II** Cases of psychiatric morbidity for each clinician

| Clinician | No. of patients | GHQ-30 | (%) | HAD scale anxiety | (%) | Depression | (%) |
|---|---|---|---|---|---|---|---|
| *Interview 1* | | | | | | | |
| A | 25 | 5 | (20) | 4 | (16) | 0 | (0) |
| B | 16 | 4 | (25) | 2 | (12) | 1 | (6) |
| C | 25 | 9 | (36) | 11 | (44) | 2 | (8) |
| D | 21 | 10 | (47) | 9 | (43) | 3 | (14) |
| E | 30 | 7 | (23) | 4 | (13) | 2 | (7) |
| *Interview 2* | | | | | | | |
| A | 18 | 4 | (22) | 2 | (11) | 0 | (11) |
| B | 11 | 6 | (54) | 3 | (27) | 1 | (27) |
| C | 31 | 4 | (13) | 5 | (16) | 2 | (16) |
| D | 19 | 7 | (37) | 6 | (31) | 2 | (31) |
| E | 22 | 1 | (4) | 4 | (17) | 1 | (17) |

**Table III** Spearman rank correlations between patients' self-reported scores and oncologists' ratings of their patients' distress

| Clinican | GHQ-30 (1) | GHQ-30 (2) | HAD scale Anxiety (1) | HAD scale Anxiety (2) | HAD scale Depression (1) | HAD scale Depression (2) |
|---|---|---|---|---|---|---|
| A | 0.07 | 0.38 | 0.20 | 0.34 | 0.18 | 0.35 |
| B | 0.61* | 0.85** | 0.47+ | 0.53 | 0.61* | 0.73* |
| C | 0.22 | 0.32+ | 0.35+ | 0.15 | 0.18 | 0.19 |
| D | 0.26 | 0.15 | 0.27 | 0.05 | 0.50* | 0.20 |
| E | 0.08 | 0.05 | 0.16 | −0.09 | −0.24 | 0.08 |

+$P<0.05$; *$P<0.01$; **$P<0.001$.

### Discussion

This study set out to assess the individual ability of oncologists to rate distress in their out-patients and investigate their levels of satisfaction in relation to handling bad news interviews. The oncologists who took part were at either consultant or senior registrar level and as senior members of staff can be said to be representative of oncologists in general. However, none had received any formal, significant undergraduate or postgraduate training in communication skills.

The level of global psychological morbidity detected by the GHQ-30 at interview 1 was 30%, later falling to 22% at interview 2. As in previous studies (Ford *et al.*, 1990; Moorey *et al.*, 1991) most of the initial morbidity comprised cases of anxiety (26% at interview 1) rather than depression (7% at interview 1). The low overall mean distress ratings for interviews 1 and 2 (5 and 3 respectively) suggest that the majority of oncologists under- rather than over-rated the distress in their patients. This is also reflected in the high mean levels of satisfaction (12 at both interviews) that they expressed with their performances. The satisfaction ratings for clinician B (the most accurate rater) were no higher or lower than those of the others.

The previously cited study (Sensky *et al.*, 1989) reported that the oncologists' ratings were all similar in distribution, but did not correlate individual oncologist ratings with patients' scores. However, in our study the oncologists' ratings of their patients' distress differed widely and the ratings of each clinician were correlated separately with the distress scores of his or her patient group.

It would appear from the percentage of cases in Table II that some clinicians had more distressed patients than others. However, this had no bearing on the mean GHQ-30 and HAD scale scores which varied little between each clinician's patient group and provided a more sensitive measure of the general distress being investigated. The fact that one clinician's ratings of patient distress consistently correlated with patients' scores indicates that the task set was not an unreasonable one and the method used was an effective test of oncologists' individual ability to assess patients' mood states. Internal consistency of all the clinicians' ratings can be demonstrated since there were two separate times of assessment (i.e. interviews 1 and 2), during which the best rater (clinician B) continued to rate accurately and the poorer raters continued to rate poorly. Furthermore, the GHQ-30 and HAD scale scores (at interviews 1 and 2) for those patients seen by clinician B differed little from those of the patients seen by the other clinicians.

Significant, negative correlations were found between clinician satisfaction ratings and corresponding clinician-reported rates of patient distress. This means that the more distressed a clinician perceived a patient to be, the less satisfied they were with the interview. No such relationship was found between clinician satisfaction ratings and patient-reported distress, although all coefficients were negative except for one, for which there was no relationship.

This lack of significant relationships is not surprising as few clinicians' distress ratings actually correlated with patient scores so it is unlikely that their overall ratings of satisfaction would do so.

A general finding of this study is that the ability to detect distress varies between different oncologists. On the whole, the oncologists under-rated the distress in their patients and consequently were usually satisfied with their handling of each interview. This is perhaps a reflection of the poor standard of training in assessment skills which doctors receive in general and reinforces the conclusions of earlier work which calls for clinicians to upgrade these skills. There is, however, evidence (Maguire, 1985) to suggest that some clinicians are better able than others to detect psychiatric distress because they are more likely to allow patients to express concerns and to pick up on other verbal and vocal cues. For example, clinicians with low identification rates tend to avoid eye contact with the patient and ask many closed questions concerning only physical symptoms. Such behaviours may prevent the patient from disclosing psychological symptoms either in words or by their tone of voice (Davenport *et al.*, 1987). The clinician may also fail to recognise postural and movement cues exhibited by the patient which may be indicative of psychological distress. There is sometimes a fear that direct probing into psychological areas will release strong emotions from a patient which the clinician will be unable to address (Buckman, 1984). The resulting reluctance of the clinician to explore this area will inevitably deter patients from disclosing any psychological problems.

Cancer clinicians freely admit that they need further training in skills which will enable them to assess accurately their patients' problems (Hopwood & Maguire, 1992). They want help in handling situations with angry patients and relatives, patients whose prognosis is uncertain and patients who deny the reality of their cancer (Maguire & Faulkner, 1988). However, experienced oncologists are often reluctant to disclose their lack of skills to their colleagues (Hopwood & Maguire, 1992). This strengthens the need for assessment and counselling skills to be an integral part of undergraduate training and for continuous evaluation and training of established medical oncologists, on an individual basis if necessary, in this important area.

Providing a model of the skills to be learned and the opportunity to practise these skills under supervision with feedback on performance has been shown to be effective in helping medical students improve and maintain their assessment skills (Maguire *et al.*, 1986). Training courses now exist which are structured to include help with personal growth and awareness, which are necessary for effective behavioural change (Bird *et al.*, 1993). These include communication skills training programmes specifically designed for senior oncologists.

We would like to thank the five clinicians who took part in this study, the Cancer Research Campaign for financial support and Patricia McHugh, who collected the data.

### References

BIRD, J., HALL, A., MAGUIRE, G.P. & HEAVEY, A. (1993). Workshops for consultants on the teaching of clinical communication skills. *Med. Educ.*, **27**, 181–185.

BUCKMAN, R. (1984). Breaking bad news: why is it still so difficult? *Br. Med. J.*, **288**, 1597–1599.

DAVENPORT, S., GOLDBERG, D. & MILLAR, T. (1987). How psychiatric disorders are missed during medical consultations. *Lancet*, **ii**, 439–441.

DEROGATIS, L.R., ABELOFF, M.D. & MCBETH, C.D. (1976). Cancer patients and their physicians in the perception of psychological symptoms. *Psychosomatics*, **17**, 197–201.

DEROGATIS, L.R., MORROW, G.R., FETTING, J., PENMAN, D., PIASETSKY, S., SCHMALE, A.M., HENRICHS, M. & CARNICKE, C.L.M. (1983). The prevalence of psychiatric disorder among cancer patients. *JAMA*, **249**, 751–757.

FALLOWFIELD, L.J., BAUM, M. & MAGUIRE, G.P. (1986). Effects of breast conservation on psychological morbidity associated with diagnosis and treatment of early breast cancer. *Br. Med. J.*, **293**, 1331–1334.

FALLOWFIELD, L.J. (1988). Psychological complications of malignant disease. *Bailliere's Clin. Oncol.*, **2**, 461–478.

FALLOWFIELD, L.J. (1993). Giving sad and bad news. *Lancet*, **341**, 476–478.

FARBER, J.M., WEINERMAN, B.H. & KUYPERS, J.A. (1984). Psychosocial distress in oncology outpatients. *J. Psychosoc. Oncol.*, **2**, 109–118.

FORD, M.F., JONES, M., SCANNELL, T., POWELL, A., COOMBES, R.C. & EVANS, C. (1990). Is psychotherapy feasible for oncology outpatients attenders selected on the basis of psychological morbidity? *Br. J. Cancer*, **62**, 624–626.

GOLDBERG, D. (1986). Use of the general health questionnaire in clinical work. *Br. Med. J.*, **293**, 1188–1189.

GOLDBERG, G. & WILLIAMS, P. (1988). *A User's Guide to the General Health Questionnaire*. NFER-Wilson: Windsor.

HALL, J.A., ROTER, D.L. & KATZ, N.R. (1988). Meta-analysis of correlates of provider behavior in medical encounters. *Med. Care*, **25**, 657–660.

HARDMAN, A., MAGUIRE, P. & CROWTHER, D. (1989). The recognition of psychiatric morbidity on a medical oncology ward. *J. Psychol. Res.*, **33**, 235–239.

HOPWOOD, P. & MAGUIRE, P. (1992). Priorities in the psychological care of cancer patients. *Int. Rev. Psych.*, **4**, 35–44.

MAGUIRE, P. (1985). Improving the detection of psychiatric problems in cancer patients. *Soc. Sci. Med.*, **20**, 819–823.

MAGUIRE, P., FAIRBAIRN, S. & FLETCHER, C. (1986). Consultation skills of young doctors. I. Benefits of feedback training in interviewing as students persist. *Br. Med. J.*, **292**, 1573–1578.

MAGUIRE, P. & FAULKNER, A. (1988). How to improve the counselling skills of doctors and nurses in cancer care. *Br. Med. J.*, **297**, 847–849.

MASSIE, M.J. & HOLLAND, J.C. (1989). Overview of normal reactions and prevalence of psychiatric disorders. In *Handbook of Psychooncology*, Holland, J.C. & Rowland, J.H. (eds) pp. 273–281. Oxford University Press: New York.

MOOREY, S. (1988). The psychological impact of cancer. In *Oncology for Nurses and Health Care Professionals*, Webb, P. (ed.) pp. 14–35. Harper & Row: London.

MOOREY, S., GREER, S., WATSON, M., GORMAN, C., ROWDEN, L., TUNMORE, R., ROBERTSON, B.M. & BLISS, J. (1991). The factor structure and factor stability of the Hospital Anxiety and Depression scale in patients with cancer. *Br. J. Psychiatry*, **158**, 255–259.

SENSKY, T., DENNEHY, M., GILBERT, A., BEGENT, R., NEWLANDS, E., RUSTIN, G. & THOMPSON, C. (1989). Physician's perceptions of anxiety and depression among their outpatients: relationships with patients' and doctors' satisfaction with their interviews. *J. R. Coll. Phys.*, **23**, 33–38.

ZIGMOND, A.S. & SNAITH, R.P. (1983). The hospital anxiety and depression scale. *Acta Psychiat. Scand.*, **67**, 370–374.